# Cross‐cultural equivalence of parental ratings of child difficulties during the pandemic: Findings from a six‐site study

**DOI:** 10.1002/mpr.1933

**Published:** 2022-08-22

**Authors:** Sarah Foley, Luca Ronchi, Serena Lecce, Xin Feng, Meingold H. M. Chan, Claire Hughes

**Affiliations:** ^1^ Moray House School of Education and Sport University of Edinburgh Edinburgh UK; ^2^ Department of Brain and Behavioural Sciences University of Pavia Pavia Italy; ^3^ Department of Human Science Ohio State University Columbus Ohio USA; ^4^ Centre for Family Research University of Cambridge Cambridge UK

**Keywords:** child psychopathology, COVID‐19 pandemic, cross‐cultural, measurement invariance

## Abstract

**Objectives:**

The Strengths and Difficulties Questionnaire (SDQ) has been shown to be invariant across informants, developmental stage and settings, but tests of cross‐cultural equivalence are limited to adolescents' self‐reports. The COVID‐19 pandemic makes this gap particularly pertinent, given the need to understand whether distinct government approaches (e.g., school closures) are uniquely associated with variability in children's psychosocial outcomes and the reliance on parents' ratings for young children.

**Methods:**

Within a Confirmatory Factor Analysis framework, we tested the cross‐cultural measurement invariance of the SDQ across six countries: Australia, China, Italy, Sweden, the United Kingdom and USA, using a sample of 1761 parents of 3‐ to 8‐year‐olds (*M* = 5.76, *SD* = 1.09).

**Results:**

A five‐factors model showed good fit to the data and partial cross‐cultural scalar invariance. In this sample, Swedish parents reported the fewest peer problems (Cohen's *d* = 0.950) and the highest prosocial scores (Cohen's *d* = 0.547), whilst British parents reported the greatest child emotional (Cohen's *d* = 0.412) and hyperactivity problems (Cohen's *d* = 0.535).

**Conclusions:**

The present results indicate that the parent‐version of the SDQ is appropriate for use and comparison across different contexts during the pandemic.

## 1 INTRODUCTION

Globally, COVID‐19 is the worst public health crisis in living memory (UNFPA, 2020); the resulting government restrictions profoundly impacted children's social life (Banerjee & Rai, 2020) and are likely to lead to long‐term negative consequences. For example, school closures have dramatically curtailed learning and social interaction opportunities for an estimated 1.5 billion children across 188 countries (Lee, 2020). Yet young children's experiences have been relatively neglected, as studies of the pandemic's socio‐emotional impact have focused on adolescents (Benner & Mistry, 2020; Stewart et al., 2022). The exceptional research with young children indicates that, compared with older children, 4‐ to 10‐year‐olds show a larger increase in mental health symptoms, with a 10% increase in children meeting clinical criteria for emotional problems (Waite et al., 2021). In line with developmental cascade models (Masten & Cicchetti, 2010), this is particularly worrying given elevated internalising and externalising problems in early childhood appear to set the stage for persistent problems across a range of domains into middle childhood and adolescence (e.g., Cyr et al., 2022; Davis et al., 2015; Kemmis‐Riggs et al., 2020). Thus, an urgent research challenge is to examine factors that account for variation in young children's adjustment during the COVID‐19 pandemic.

Do cultural factors and COVID‐19 related policies (i.e., school closure, lockdowns) mitigate or exacerbate adverse consequences of the pandemic on children's emotional and behavioural adjustment (Fegert et al., 2020)? Existing studies (e.g., Geweniger et al., 2022; Serra et al., 2022) have typically adopted single‐site designs (see Singh et al., 2020 for a review), raising questions about the generalisability of findings and precluding analysis of moderating effects of governmental policies. An important prerequisite for meaningful cross‐cultural comparisons is to test whether instruments used to assess child behavioural and emotional problems show ‘measurement invariance’, and hence indicate cross‐cultural equivalence across groups (Putnick & Bornstein, 2016). Where measurement invariance does not hold, cross‐national differences in ratings may reflect measurement biases rather than genuine differences in mental health rates (Goodman et al., 2012). Considering markedly different COVID‐19 experiences, not limited to contrasts in rates of infection, mortality, and restrictions, we applied tests of measurement invariance to establish the suitability of cross‐cultural comparisons of children's adjustment difficulties during the pandemic.

### 1.1 Cross‐cultural measurement invariance in assessments of child psychopathology

Rates of child psychopathology vary across the globe (Achenbach et al., 2012). In a systematic review (*K* = 41), Stevanovic et al. (2017) examined: (i) how many studies test the cross‐cultural equivalence of scales assessing children and young people's mental health; and (ii) whether these studies typically demonstrate measurement invariance. Full measurement invariance means that the entire distribution of expected observed scores given trait levels is the same across groups (Molenaar & Borsboom, 2013). As full measurement invariance is difficult to establish, factorial invariance is commonly used to address the equivalence of measurement models across groups and times (Chiorri et al., 2016; Murray et al., 2019; Ortuno‐Sierra et al., 2015). Factorial invariance is typically examined within a confirmatory factor analytic (CFA) framework and tests whether the mean and variance of observed score distributions, given latent trait levels, are the same across groups (Molenaar & Borsboom, 2013). There are different levels of invariance across groups: *configural invariance* indicates the same factor structure (i.e., scale items belong to the same factors). *Metric invariance*, also known as weak measurement invariance, refers to the equality of factor loadings (i.e., a unit increase in the latent variable is associated with equal increases in corresponding indicators across groups). *Scalar invariance*, also known as strong measurement invariance, refers to the equality of intercepts or item thresholds (i.e., respondents with the same level of the latent construct across groups have the same expected score on the measured indicators). Establishing scalar invariance is generally considered sufficient for conducting appropriate between‐group comparisons of latent means (Chiorri et al., 2016). Furthermore, it is common for researchers to demonstrate *partial* invariance, where a subset of items fails to reach the criteria for full metric and/or scalar invariance (Putnick & Bornstein, 2016). Partial invariance modelling solutions allow estimates for the non‐invariant parameters to vary across groups while keeping estimates of invariant parameters constrained to equality across groups (Dong & Dumas, 2020). Simulation studies demonstrate that partial scalar invariance is sufficient to conduct appropriate comparison of latent means, with 20%–80% non‐invariant items permitted (Pokropek et al., 2019; Steinmetz, 2013).

Stevanovic et al. (2017) concluded that while some symptom‐specific scales show full or partial scalar invariance across multiple sites (e.g., the Revised Children's Manifest Anxiety Scale; Reynolds & Richmond, 2000), scales assessing general psychopathology yield more mixed results. Specifically, there was inconsistent evidence from the four studies testing the self‐report version of the Strengths and Difficulties Questionnaire (SDQ) (Goodman, 1997) and no evidence of factorial invariance across the five studies examining the parent version of the Child Behaviour Checklist (Achenbach & Ruffle, 2000). This review also highlighted the overall paucity of research into the cross‐cultural equivalence of parent reports, despite their importance for research with young children (i.e., 3–7 years) who are not reliably able to complete self‐report psychological questionnaires (Muris et al., 2004). Furthermore, constraining the generalisability of emerging findings, most studies of cross‐cultural factorial invariance either: (i) involve relatively small numbers of sites (36/41 studies included just 2 to 4 groups; Stevanovic et al., 2017), or (ii) compare different cultural groups within a country (e.g., Varela et al., 2008). Addressing these limitations, we sought to assess whether parental ratings of child adjustment difficulties during the COVID‐19 pandemic showed factorial invariance and were, therefore, comparable across six different study sites.

### 1.2 Tests of measurement invariance of the strengths and Difficulties Questionnaire

Globally, the SDQ is one of the most widely used tools for measuring children's social, emotional, and behavioural problems (Goodman, 1997, 1999), with translations into over 60 languages (Achenbach et al., 2008). The scale consists of five 5‐item scales, of which four probe difficulties (emotional symptoms, conduct problems, hyperactivity–inattention, and peer problems) and one probes strengths (prosocial behaviour). The SDQ has good psychometric properties: both the parent and teacher versions show validity evidence based on internal structure (i.e., internal consistency), test–retest reliability of the scores, and inter‐rater agreement on the scores (Stone et al., 2010). Recent analyses of nationally representative data from the United Kingdom (UK) demonstrated that parent and teacher SDQ ratings show measurement invariance across the broad developmental period from preschool to adolescence (Murray et al., 2021a, 2021b). Support for measurement invariance of the parent version of the SDQ has also been found across informants (Chiorri et al., 2016; Rogge et al., 2018), community and clinical samples (Smits et al., 2016), gender and age of the child (Palmieri & Smith, 2007) and parent education level (Stone et al., 2013).

Regarding validity evidence of the internal structure of the SDQ, several theoretically plausible factor structures have been proposed (see Chiorri et al., 2016 for a review). The original five‐factor solution is the most widely replicated for the parent‐report version of the SDQ (Gomez & Stavropoulos, 2020; Stone et al., 2010), demonstrating the best model fit and strong links with corresponding psychiatric diagnoses (de la Cruz et al., 2017). A three‐factor model has also received some support (e.g., Dickey and Blumberg (2004) found a broader distinction between prosocial, internalizing and externalizing behaviours provided the best fitting model in a sample of parents with 4‐ to 17‐years old children in the US). Supporting a higher‐order conceptualization of internalizing/externalizing SDQ subscales, Goodman, Lamping, and Ploubidis (2010) found that both the original five‐factor model and a second‐order model, with additional internalizing and externalizing factors, had adequate fit in a representative sample of 5‐ to 16‐year‐olds in the UK.

Finally, it is worth noting that some studies support the original five‐factor model compared to a less conservative six factor model that includes an additional method factor accounting for error covariances among the 10 items describing positive behaviours (Palmieri & Smith, 2007). It is unclear whether this six‐factor model really provides a better conceptualization of the instrument than the original five‐factor model (Chiorri et al., 2016; Gomez & Stavropoulos, 2020; McCrory & Layte, 2012).

Research examining cross‐cultural measurement invariance of the SDQ has largely been restricted to the self‐report version, which is suitable for children over 11 years old (Duinhof et al., 2020; Essau et al., 2012; Ortuño‐Sierra et al., 2015; Stevanovic et al., 2015). Two of these studies provide no evidence for the cross‐cultural measurement invariance of adolescent ratings of the SDQ, respectively finding configural (Essau et al., 2012) and non‐invariance (Stevanovic et al., 2015). In contrast, two other studies (Duinhof et al., 2020; Ortuno‐Sierra et al., 2015) report that adolescent SDQ ratings show partial measurement invariance (non‐invariant loadings), while nationally representative data from 33, 233 adolescents across seven countries also support the suitability of cross‐cultural comparisons (Duinhof et al., 2020), although it was necessary to drop specific (reversed) items (e.g., *generally obedient*). Such inconsistency might suggest that for late adolescence the SDQ has different measurement properties (i.e., over 17; Murray et al., 2021a).

### 1.3 Parents and pandemics: Extending existing research into the cross‐cultural measurement invariance of the SDQ

Zwirs et al. (2011) demonstrated full strong measurement invariance for teacher SDQ ratings of school‐aged Native Dutch, Moroccan, Turkish and Suinamese children in the Netherlands. Another study gathered parent, teacher and child SDQ ratings from over 14,000 5 to 16‐year‐olds in England and demonstrated that the five‐factor model (with two additional second‐order factors reflecting internalising and externalising problems) was invariant across informants as well as ethnic groups (i.e., Indian and White British) (Goodman, Patel, & Leon, 2010). To our knowledge, however, researchers have yet to examine across‐country measurement equivalence for the parent version of the SDQ.

Five reasons make this gap important. First, mass school closures have made it necessary for researchers and clinicians to rely on parental ratings for young children. In this context, brevity makes the SDQ the ideal choice of instrument for gathering ratings from time‐poor parents. Second, while the SDQ has already been widely used in studies of the impact of COVID‐19 (e.g., Liu et al., 2021; Waite et al., 2021), its suitability in the context of the pandemic requires testing. For instance, social distancing measures are likely to affect the relevance of items assessing difficulties interacting with other children, which will in turn impact the factor structure of the underlying constructs. Third, the pandemic has the potential to heighten cross‐cultural differences between countries. While the outbreak of COVID‐19 has had a devastating impact across the globe, between‐ and within‐country variation in health, economic and social side‐effects are also striking, yet their relative magnitudes have yet to be established. Fourth, testing for cross‐cultural measurement invariance of parent SDQ ratings will be useful in the advent of future pandemics, natural/economic disasters, and war. Finally, testing measurement invariance can contribute to larger theoretical debates about the universality of mental health constructs. Specifically, lack of measurement invariance may preclude meaningful cross‐cultural comparisons but highlights interesting differences in the ways such constructs are conceptualised or manifest across cultures.

In sum, the present study aimed to extend existing research with the SDQ by testing for structural and factorial invariance for parental ratings of 3‐ to 8‐year‐old children's adjustment problems across six geographically and culturally different sites: Australia, China, Italy, Sweden, the UK and United States of America. Crucially, if at least partial factorial invariance was established, our secondary aim was to compare mean parents' ratings of child adjustment across these distinct contexts. We anticipated the five‐factor model of SDQ scores would provide a good fit to our data for each country and be invariant across cultures, at least at the configural level. However, we adopted an exploratory perspective when pursuing tests of metric or scalar invariance.

## 2 METHODS

### 2.1 Participants and procedure

The present study capitalised on data collected as part of a large‐scale online survey, conducted between April and July 2020, to examine young children's development and family adjustment in the COVID‐19 pandemic. A sample of 2516 parents with one or more children between the ages of 3.00–7.99 years old were recruited via social media and mailing lists in Australia, China, Italy, Sweden, the UK, and the United States of America (USA). This sample of parents reflects 58% of the 4329 respondents who started the questionnaire, specifically 55 respondents were not eligible to participate as they indicated they had a major psychiatric problem or learning difficulty and a further 1758 did not complete the survey beyond providing consent. The survey took approximately 45 minutes to complete. In Spring 2020, these six countries were at different points of virus transmission (infections in China preceded cases in the other sites). Furthermore, when completing the SDQ, parents within each country were experiencing different levels of active restrictions linked to virus transmission (see Hale et al., 2020). The survey was developed, translated from English and hosted on Qualtrics. The specific validated version of the SDQ questionnaire was adopted in each country. Ethical committees from each site approved the study protocol.

Participants with missing data on all SDQ items were excluded. Excluded cases did not differ from included cases in terms of children's gender, *χ* ^*2*^(1) = 0.596, *p* = 0.46, target child's age, *t* = 0.839, *p* = 0.40, and responding parent's age, *t* = 0.011, *p* = 0.99. However, excluded cases were less highly educated (67% had a degree) than included cases (74% had a degree), *χ* ^*2*^(1) = 10.690, *p* = 0.001. The final sample comprised a total of 1761 respondents, of whom 6.2% were in Australia (*n* = 109), 13.3% were in China (*n* = 234), 7.4% were in Italy (*n* = 130), 32.2% were in Sweden (*n* = 566), 29% were in the UK (*n* = 512) and 11.9% were in the USA (*n* = 210). As illustrated in Table 1, respondents were typically female caregivers (81.2% female and 8.6% male, 10.2% prefer not to say), aged between 21 and 65 years old (*M*age = 37.14, *SD* = 6.06 years), and highly educated (63.1% undergraduate degree or higher). Of the 50% who reported their ethnicity, 50% identified as White, 29% as Asian and 21% as having mixed or multiple ethnicities. Just under half (48.2%) the children were female and they ranged from 3.00 to 7.99 years in age (*M* = 5.76; *SD* = 1.09). The proportion of males and females did not differ across countries, *χ* ^*2*^(5) = 4.92, *p* = 0.43. Modest differences were found by countries in children's age, *F*(51,755) = 21.68, *p* < 0.01, partial *η* ^2^ = 0.06. Overall, Chinese children were the youngest, with a mean age of 5.33 (*SD* = 0.97) corresponding to 0.4 *SD* below the grand mean and Australian children were the oldest, with a mean age of 6.24 (*SD* = 1.04) corresponding to 0.4 *SD* above the grand mean.

**TABLE 1:** Family demographics by site

	Overall	UK	Italy	China	Australia	USA	Sweden
Parent							
Age (years)	37.14	37.45	39.69	34.70	38.90	37.50	36.81
*M* (*SD*)	(6.06)	(7.13)	(5.14)	(5.79)	(5.46)	(6.51)	(4.74)
Education % degree	63%	67%	60%	19%^a^	74%	83%	7069%
Child							
Age (years)	5.76	6.04	5.79	5.33	6.25	5.64	5.62
*M* (*SD*)	(1.09)	(1.06)	(1.13)	(0.97)	(1.05)	(1.02)	(1.09)
Sex % female	48%	46%	46%	46%	55%	48%	51%

*Note*: ^a^ 75% missing.

### 2.2 Instrument

Participants completed the parents' version of the Strengths and Difficulties Questionnaire (SDQ: Goodman, 1997). It consists of five subscales, each with five items rated on a three‐point scale (not true, somewhat true, certainly true). Four subscales relate to child difficulties: emotional problems, peer problems, conduct problems and hyperactivity, and one subscale concerns prosocial behaviour. The original English version was used in the English, Australian and American samples (Goodman, 1997), with nationally validated versions of the SDQ administered in Italy (Marzocchi et al., 2004), China (Du et al., 2008) and Sweden (Malmberg et al., 2003).

### 2.3 Analysis plan

First, we conducted a series of CFAs to evaluate the goodness of fit of the original SDQ five factor model to our data and to compare this model with alternative factor models. Based on the literature, we compared the original five‐factor solution to (a) a nested (more parsimonious) three factor model with latent factors for externalising problems (i.e., conduct problems and hyperactivity), internalising problems (i.e., emotional and peer problems), and prosociality (Dickey & Blumberg, 2004) and to (b) a less parsimonious six factor model including the original five factors plus the additional method factor accounting for error covariance among positive worded items (McCrory & Layte, 2012). To be thorough we also evaluated other existing SDQ models (i.e., five factor with one higher order factor model, two factor model and single factor model), despite these models having received less support in the literature (e.g., Stone et al., 2010). Prior to this, we screened the data to check for lack of variation due to the COVID‐19 related restrictions.

Second, we used multiple‐group categorical confirmatory factor analysis (MG‐CFA) to test for factorial invariance of the SDQ across sites. This involves imposing increasingly stringent equality restraints to the measurement model across sites and testing the change in model fit of these nested models (Brown, 2015). A significant decrease in model fit indicates that at least one of the measurement model's constrained parameters is non‐invariant in at least one site and should be free to vary to achieve a partial factorial invariance solution (Jung & Yoon, 2016). Non‐invariant loadings or thresholds are released one at a time starting from the one leading to the greatest improvement in model fit (i.e., highest model modification indices). We used mean‐ and variance‐adjusted weighted least squares (WLSMV) estimator with Delta parameterisation (Muthén & Asparouhov, 2002). We set scaling and identification constraints of invariance models to the values suggested by Muthén and Muthén (1998‐2012). In the configural model: (a) item thresholds and factor loadings were free to vary across groups, (b) the first item for each latent factor was fixed at one, with latent factor variances being free to vary across groups, (c) scale factors were fixed at one in all groups and factor means were fixed at zero in all groups. To test metric invariance at the second step, factor loadings and latent factor variances were constrained to equality. In this second step, scale factors were still fixed at one in all groups for identification issues. Third, equality of thresholds was added to test for scalar invariance. In this scalar model, scale factors were fixed at one and zero, respectively, in the first group only. Next, starting from the final (full or partially) invariant modelling solution, the latent scores' reliability was computed as the McDonald's Omega Coefficient (*ω*) (McDonald, 1999) and latent means invariance was tested by fixing them to zero in all groups. The sample size within each of the sites exceeded 100 cases, satisfying general power recommendations for conducting MG‐CFA (Kline, 2016; Wang et al., 2013). We evaluated model fit using three primary criteria: Comparative Fit Index (CFI) > 0.90, Tucker Lewis Index (TLI) > 0.90, Root Mean Square Error of Approximation (RMSEA) < 0.08 (Brown, 2015). Due to Δ*χ* ^2^ sensitivity to sample size, nested model comparisons were deemed as nonsignificant using the following criteria: ΔCFI > −0.010 and ΔRMSEA <0.010 (Chen, 2007; Cheung & Rensvold, 2002; Rutkowski & Svetina, 2017). Materials and analysis code for this study are available from the corresponding author.

## 3 RESULTS

Data screening (see supplementary Table S1 for details) indicated zero ratings for more than 83% of responses to one item from the peer problems subscale (*bullied by other children*) and two from the conduct problems subscale (*fights with other children*; *steals from home or school*). Mindful of theoretical and statistical justification for model specification (Byrne, 2012), as COVID‐19 related restrictions may have made these items unsuitable and they were excluded from further analyses.

Supplementary Table S2 shows the goodness‐of‐fit indices comparing the alternative SDQ factor structures in the overall sample and across sites. The original five‐factor model showed acceptable fit to the data and significantly better goodness‐of‐fit indices compared to each of the more parsimonious solutions (i.e., five factor with one higher‐order factor model; three factor model, two factor model and single factor model). The six‐factor model showed the best fit to the data in the overall sample and within individual sites. Notwithstanding, the pattern of item loadings for the six‐factor model was heterogeneous across countries and included several non‐significant loadings on the prosocial and peer problems factors (e.g., *Often volunteers to help others*, *rather solitary*, *tends to play alone*). Moreover, three positive‐worded items (i.e., *Considerate of other people's feelings*, *shares readily with other children* and *sees tasks through to the end*) did not significantly cross‐load onto the method factor in Italy, Australia and China, respectively. In contrast, the pattern of loadings was more consistent across groups for the original five‐factor model. Only one peer problems scale item (*gets on better with adults than peers*) did not load significantly in China or Australia (see supplementary Table S3 for detailed information about factorial loadings and factors correlations for the five factor model across sites). Given these results, we retained the five‐factor model as a parsimonious common measurement model for factorial invariance testing.

### 3.1 Cross‐cultural invariance

We applied a MG‐CFA framework to test for factorial invariance of the five‐factor SDQ solution (see Table 2 for goodness‐of‐fit indices and nested model comparisons). The configural invariant model had an acceptable fit to the data, RMSEA = 0.064 [95%confidence Intervals (CI) 0.060, 0.067], CFI = 0.922, TLI = 0.909. As reported above, only one item (*gets on better with adults than peers*) failed to load significantly onto the peer problems latent factor for the Chinese and Australian samples. Given that configural invariance was prevented by just one item we preferred to redefine the construct (i.e., omit the item and retest the model) rather than discontinue invariance and group difference testing (Putnick & Bornstein, 2016). After omitting this item, the configural model continued to show an acceptable fit to the data, RMSEA = 0.064 [95%CI 0.060, 0.067], CFI = 0.928, TLI = 0.915, indicating an equal pattern of loadings across sites. The next model constraining factor loadings to be equal across sites (i.e., metric invariance) showed an acceptable fit to the data, RMSEA = 0.064 [95%CI 0.061, 0.068], CFI = 0.920, TLI = 0.915. The lack of significant deterioration in model fit compared to the less parsimonious configural model suggested that metric invariance held, ΔCFI = −0.008, ΔRMSEA = 0.000. Finally, our model testing for scalar invariance, by constraining item thresholds to equality, had an acceptable fit to the data, RMSEA = 0.067 [95%CI 0.064, 0.070], CFI = 0.908, TLI = 0.908. Nevertheless, while the ΔRMSEA <0.010 criterion was met, the ΔCFI marginally exceeded the proposed cut‐off (ΔCFI = 0.012). Modification indices indicated that the first threshold for item 15 (i.e., *easily distracted*, *concentration wanders*) should be released in the Swedish group. This resulted in acceptable model fit, CFI = 0.911, TLI = 0.911, RMSEA = 0.066 [95%CI 0.064, 0.070], with both ΔRMSEA and ΔCFI within the pre‐defined accepted criteria, ΔCFI = −0.009, ΔRMSEA = 0.002 (see supplementary Table S4 for detailed information about factorial loadings, item thresholds and latent means). This final model supports partial factorial invariance up to the scalar level, with only one item threshold being invariant in only one site. Reliability coefficients (*ω*) for the SDQ latent factors' scores based on this final model, and therefore valid for all the countries, were 0.85 for emotion, 0.67 for conduct, 0.86 for hyperactivity, 0.70 for peer and 0.84 for prosociality.

**TABLE 2:** Nested models comparisons for the factorial invariance analysis

Five‐factor model	CFI	TLI	RMSEA [950% CI]	χ^2^	df	ΔCFI	ΔRMSEA
Configural invariance^a^	0.922	0.909	0.064 [0.060, 0.067]	2615.947	1194	‐	‐
Configural invariance^b^	0.928	0.915	0.064 [0.061, 0.068]	2378.710	1074	‐	‐
Metric invariance	0.920	0.915	0.064 [0.061, 0.068]	2618.514	1179	0.008	0.000
Scalar invariance	0.908	0.908	0.067 [0.064, 0.070]	2924.620	1259	0.012*	0.003
Partial scalar invariance	0.911	0.911	0.066 [0.063, 0.069]	2873.180	1258	0.009	0.002
Latent means constraints	0.878	0.880	0.077 [0.074, 0.080]	3490.064	1283	0.033*	0.011*
Partial latent means constraints	0.908	0.910	0.067 [0.063, 0.070]	2939.656	1279	0.003	0.001

*Note*: *Exceed cut‐off criteria (i.e., ΔCFI > −.010 and ΔRMSEA <.010).

Abbreviations: CFI, Cumulative Fit Index; CI, Confidence Intervals; RMSEA, Root Mean Square Error of Approximation; TLI, Tucker Lewis Index.

^a^ Configural model including item 23.

^b^ Configural model excluding item 23.

### 3.2 Mean cross‐cultural differences in the strengths and Difficulties Questionnaire

**FIGURE 1:**
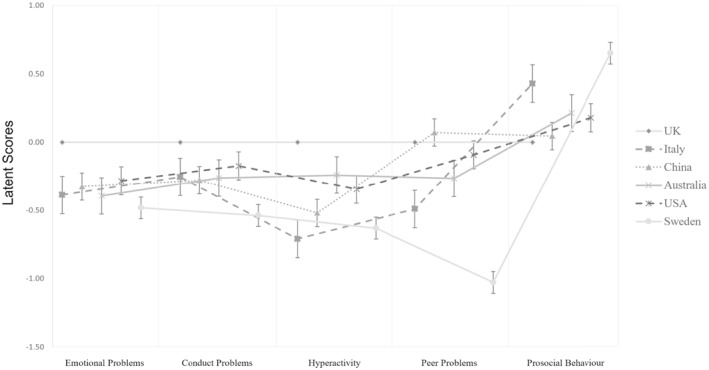
Latent Factor Means Across Sites. Latent means and standard errors of Strengths and Difficulties Questionnaire (SDQ) subscales by sites based on the final partially scalar invariant solution. In this model latent means are fixed to zero in the reference group only (i.e., the United Kingdom (UK)) for model identification purposes (see Table S4). See the results section and Table S5 for results of latent means comparisons

To compare latent factor means, we constrained them to zero in all groups, which significantly decreased model fit, ΔCFI = −0.033, ΔRMSEA = 0.011. Inspection of the model modification indexes showed that latent mean constraints should be released for the hyperactivity and emotion problems factors in the British group and the prosocial behaviour and peer problems factors in the Swedish group. The final invariant model with latent means fixed to equality except for the four listed above showed an acceptable fit to the data, CFI = 0.908, TLI = 0.910, RMSEA = 0.067 [95%CI 0.063, 0.070], with both ΔRMSEA and ΔCFI within the accepted criteria, ΔCFI = −0.003, ΔRMSEA = 0.001 (see supplementary Table S5 for detailed information about factorial loadings, item thresholds and partially constrained latent means). On average, British respondents scored 0.535 *SD* higher than other respondents on the hyperactivity latent factor and 0.412 *SD* higher on the emotion problems latent factor, while Swedish respondents scored 0.547 *SD* higher than other respondents on the prosocial behaviour latent factor and 0.950 *SD* lower on the peer problems latent factor(see Figure 1).

## 4 DISCUSSION

Early in the COVID‐19 pandemic, 2516 parents of 3‐ to 8‐year‐olds took part in an online survey to explore family life and child adjustment across six countries with diverse national responses (Australia, China, Italy, Sweden, the UK and USA). Mindful of the need for our cross‐cultural comparisons to be meaningful and to aid interpretations of between‐site comparisons of single‐site research, we tested the cross‐cultural structural and measurement equivalence of parent ratings of their children's strengths and difficulties (SDQ). Our analyses advance the field in three ways. First, supporting existing accounts that highlight the multi‐dimensional nature of children's adjustment problems, the original five‐factor model, reflecting four difficulties and one strength dimension, provided a good and parsimonious fit to the data. Second, partial scalar invariance across the six countries supports the suitability of using the parent‐version of the SDQ across different contexts during the pandemic. Third, Swedish parents reported the lowest levels of child peer problems and the highest level of child prosocial behaviours, whilst British parents reported the highest levels of child emotional and hyperactivity problems.

### 4.1 SDQ structure

Using a nationally representative data set of parental SDQ ratings for 10, 207 children, Murray et al. (2021a) have demonstrated gender and longitudinal invariance for the five‐dimensional strengths and difficulties structure for children seen at ages 5, 7, 11 and 14. While the SDQ has been translated into over 60 languages (https://www.sdqinfo.org), group comparisons using measures developed and validated in one cultural context may yield spurious differences (Chen, 2008). Extending previous examinations of cross‐cultural invariance for the adolescent self‐report SDQ, this study examined cross‐cultural invariance for the parent report version. Initial descriptive analyses showed floor effects for three items (exposure to bullying, fighting with peers, stealing) that were subsequently removed from the model. In the pandemic, these items may have been difficult for parents to answer, given mass school closures and social distancing measures. Note there is a precedent for dropping SDQ items, for example partial measurement invariance across seven European countries was established after dropping the five positively worded items (Duinhof et al., 2020).

Adolescent‐focused studies have yielded mixed responses regarding the structure of the self‐report SDQ (Duinhof et al., 2020; Essau et al., 2012; Ortuno‐sierra et al., 2015). However, consistent with other parent‐report studies, we found support for a five‐factor solution. While at first glance the six‐factor solution appeared to have the best fit to the data, closer inspection of the item loadings highlighted several items did not load onto their respective dimensions. Moreover, associations between the method and the five child dimensions add to the complexity of interpreting results (Eid, 2000; Marsh & Grayson, 1995). Thus, our five‐factor solution also had the advantage of greater interpretability. Echoing results of previous studies (e.g., Ortuno‐Sierra et al., 2015), the emotion, hyperactivity and prosociality subscales showed good reliability of the scores, and the peer subscale showed acceptable reliability (0.85, 0.85. 0.84 and 0.70 respectively). The conduct subscale showed modest reliability (0.67), which may reflect the reduced number of items (Brown, 2015; Tabachnick & Fidell, 2013).

### 4.2 Measurement invariance during the pandemic

Reflecting on existing research into the psychological effects of the pandemic, Demkowicz et al. (2021) identified the establishment of measurement invariance as an urgent challenge for mental health researchers, noting that this is a prerequisite for meaningful comparisons. Responding to this challenge, Caycho‐Rodríguez et al. (2021) found partial scalar invariance for a fear of COVID‐19 scale. Likewise, using data from adult employees working in China, Germany and the USA (*N* = 2351), Tang et al. (2021) reported metric invariance for measures of flourishing during the pandemic.

Extending the developmental scope of cross‐cultural comparisons of children's adjustment during times of crisis, our findings demonstrate that parental ratings of child adjustment in the pandemic show across‐site measurement invariance. Our analysis included six sites that represented different points in the trajectory of COVID‐19 transmission and associated government restrictions (Hale et al., 2020). Demonstrating measurement invariance during these conditions is therefore reassuring with regards to the SDQ's robustness and utility. In addition, previous tests of the SDQ's developmental, informant, and cross‐cultural measurement invariance have relied upon traditional pen and paper data collection. Though they have their drawbacks, online methods may reduce social desirability bias. However, their appropriateness is often assumed rather than tested. Our findings increase confidence in the similarity of psychometric properties of traditional and online formats (see also Seward et al., 2017).

### 4.3 Site contrasts in parental SDQ scores

Demonstrating across‐site equivalence of parental ratings on the SDQ strengthens the suitability of our cross‐site comparisons. Murray et al. (2021b) demonstrated between‐informant scalar measurement invariance and, highlighting the potential importance of context effects, showed that teachers reported lower mean levels of child prosociality, hyperactivity, emotional and conduct problems than did parents. In the current study, the cross‐cultural differences seem to indicate an impact of social restrictions on child adjustment, in that Swedish parents reported better child adjustment (i.e., fewer peer problems, greater prosocial behaviour) than did parents in the other five sites. This contrast directly mirrors a related between‐site contrast in the extent to which children's daily lives were disrupted by the pandemic, as Sweden was the only site in which nurseries and schools remained open throughout the pandemic. This view fits with a growing body of research demonstrating the widespread impact of school closure on children's psychological health (e.g., Chaabane et al., 2021).

Echoing longitudinal findings that suggest the pandemic has led to elevated levels of depressive symptoms among British 7 to 11‐year‐old children (Bignardi et al., 2020), in our international study, British parents reported greater child emotion problems and hyperactivity than did parents in the other five sites. This contrast may hinge on the adverse effects of parental mental health problems on child adjustment: in a recent cross‐cultural study, adults in the UK reported higher levels of fear about the pandemic than did adults in other sites (Dryhurst et al., 2020). Thus, the impact of family disruption on parental mental health may mediate the impact of the pandemic on child adjustment (see Foley et al., 2022).

### 4.4 Limitations and conclusions

In common with other online survey studies conducted during the pandemic, several methodological limitations constrain our findings. Young children have been largely overlooked in studies of the mental health impact of the pandemic, with multiple caring responsibilities and work demands making research participation a low priority for many families. This may be especially true for less affluent families for whom the burden of the pandemic has been particularly severe (Ravens‐Sieberer et al., 2021; Wanberg et al., 2020) or in the context of other existing social inequalities (e.g., Jones et al., 2022). Perhaps reflecting this, our sample was unrepresentative (predominantly educated and affluent) in nature, such that more research is needed to test whether our comparative findings generalise to socially diverse samples. Nevertheless, it is striking that Swedish children displayed fewer peer problems and greater prosociality than children in the other five sites. Future longitudinal research will enable researchers to test whether these findings hold across time and generalise to different contexts.

Despite these constraints, it is worth recalling that our study's main contribution to the field lies in demonstrating the cross‐cultural equivalence of parental SDQ ratings of child adjustment, even during the pandemic. A heavy reliance on single‐site studies limits developmental accounts of the impact of the pandemic on children (Benner & Mistry, 2020). Thus, demonstrating that groups of parents from distinct cultures provide equivalent ratings of child adjustment provides a valuable platform for future research. Such investigations should go beyond documenting differences to identifying the extent to which risk and protective factors are also culturally universal. Given the SDQ is widely used to track the impact of psychosocial interventions, establishing the cross‐cultural equivalence of the SDQ will be especially useful for comparing the effectiveness of programmes across sites.

## AUTHOR CONTRIBUTIONS

Sarah Foley: Conceptualization: Equal; Data curation: Lead; Investigation: supporting; Methodology: Equal; Project administration: Equal; Resources: Supporting; Writing – original draft: Lead. Luca Ronchi (Conceptualization: Equal; Formal analysis: Lead; Methodology: Equal; Project administration: Equal; Resources: Supporting; Writing – original draft: Lead). Serena Lecce: Conceptualization: Equal; Investigation: supporting; Supervision: Supporting; Resources: Equal; Writing – review & editing: Equal. Xin Feng: Investigation: supporting; Writing – review & editing: Equal. Meingold H. M. Chan Data curation: supporting; Investigation: supporting; Writing – review & editing: Equal. Claire Hughes Conceptualization: Equal; Funding acquisition: Lead; Methodology: Lead; Project administration: Lead; Resources: Equal; Supervision: Lead; Writing – review & editing: Equal.

## CONFLICT OF INTEREST

The author declares that there is no conflict of interest that could be perceived as prejudicing the impartiality of the research reported.

## Supporting information

Supporting Information S1

## Data Availability

The data that support the findings of this study are openly available in ‘OSF repository’ at https://doi.org/10.17605/OSF.IO/P3TJH.
