# Supplementary material for: Cross‐cultural equivalence of parental ratings of child difficulties during the pandemic: Findings from a six‐site study
Source: Int J Methods Psychiatr Res. 2022 Aug 22;32(1):e1933. doi: 10.1002/mpr.1933 (PMC9976603; doi:10.1002/mpr.1933)
Supplement: Supplementary file 1 — Supporting Information S1 [file MPR-32-e1933-s001.docx]

Table S1. *Descriptives for SDQ Items, Subscales and Total Difficulties Score in The Overall Sample and Across Sites*

| Scale | Item | Participants % | Overall | UK | Italy | China | Australia | USA | Sweden |
| --- | --- | --- | --- | --- | --- | --- | --- | --- | --- |
| **Emotional problems** | Often complains of headaches, stomach-aches or sickness | Not true | 70.3 | 67.8 | 79.8 | 77.8 | 74.1 | 67.6 | 67.5 |
|  |  | Somewhat true | 23.9 | 22.0 | 17.8 | 21.7 | 20.4 | 26.7 | 27.4 |
|  |  | Certainly true | 5.9 | 10.2 | 2.3 | 0.4 | 5.6 | 5.7 | 5.1 |
|  | Many worries, often seems worried | Not true | 58.4 | 48.9 | 67.7 | 63.9 | 62.4 | 58.1 | 62.0 |
|  |  | Somewhat true | 32.1 | 37.3 | 22.1 | 31.3 | 29.4 | 33.8 | 29.9 |
|  |  | Certainly true | 9.5 | 13.8 | 10.2 | 4.9 | 8.3 | 8.1 | 8.1 |
|  | Often unhappy, down-hearted or tearful | Not true | 69.0 | 61.4 | 68.8 | 60.7 | 70.6 | 72.3 | 77.7 |
|  |  | Somewhat true | 25.9 | 30.5 | 22.7 | 37.1 | 23.9 | 21.5 | 19.8 |
|  |  | Certainly true | 5.1 | 8.1 | 8.6 | 2.2 | 5.5 | 6.2 | 2.5 |
|  | Nervous or clingy in new situations, easily loses confidence | Not true | 39.3 | 35.5 | 44.4 | 23.5 | 45.9 | 36.2 | 47.7 |
|  |  | Somewhat true | 43.0 | 40.3 | 34.1 | 63.0 | 33.9 | 44.8 | 40.3 |
|  |  | Certainly true | 17.8 | 24.2 | 21.4 | 13.5 | 20.2 | 19.1 | 12.0 |
|  | Many fears, easily scared | Not true | 51.8 | 46.4 | 54.0 | 37.3 | 55.1 | 52.6 | 61.1 |
|  |  | Somewhat true | 36.5 | 36.1 | 35.5 | 55.3 | 37.6 | 35.9 | 29.7 |
|  |  | Certainly true | 11.6 | 17.6 | 10.5 | 7.5 | 7.3 | 11.5 | 9.2 |
|  | **Total score** | **M** | **2.60** | **3.12** | **2.39** | **2.64** | **2.34** | **2.65** | **2.21** |
|  |  | **SD** | **2.28** | **2.57** | **2.19** | **1.62** | **2.21** | **2.26** | **2.19** |
| **Conduct problems** | Often has temper tantrums or hot tempers | Not true | 35.4 | 26.8 | 39.5 | 36.8 | 34.9 | 31.1 | 43.5 |
|  |  | Somewhat true | 40.9 | 38.2 | 39.5 | 54.6 | 43.1 | 37.8 | 38.7 |
|  |  | Certainly true | 23.7 | 35.0 | 20.9 | 8.7 | 22.0 | 31.1 | 17.8 |
|  | Generally obedient, usually does what adults request | Not true | 31.6 | 28.2 | 29.7 | 18.7 | 34.9 | 29.7 | 40.5 |
|  |  | Somewhat true | 55.1 | 55.1 | 56.3 | 67.8 | 50.5 | 58.9 | 49.1 |
|  |  | Certainly true | 13.3 | 16.7 | 14.1 | 13.5 | 14.7 | 11.5 | 10.4 |
|  | Often fights with other children or bullies | Not true | 83.4 | 85.4 | 76.0 | 71.3 | 77.1 | 82.8 | 89.8 |
|  |  | Somewhat true | 14.3 | 12.3 | 20.0 | 27.0 | 21.1 | 13.4 | 8.8 |
|  |  | Certainly true | 2.2 | 2.4 | 4.0 | 1.7 | 1.8 | 3.8 | 1.4 |
|  | Often lies or cheats | Not true | 69.3 | 65.1 | 64.8 | 70.4 | 66.4 | 67.5 | 74.9 |
|  |  | Somewhat true | 26.8 | 29.2 | 27.3 | 26.9 | 29.9 | 28.2 | 23.3 |
|  |  | Certainly true | 3.9 | 5.7 | 7.8 | 2.7 | 3.7 | 4.3 | 1.8 |
|  | Steals from home, school, or elsewhere | Not true | 94.1 | 94.4 | 97.7 | 91.2 | 95.4 | 91.9 | 94.7 |
|  |  | Somewhat true | 4.7 | 4.2 | 1.6 | 7.9 | 1.8 | 5.3 | 5.0 |
|  |  | Certainly true | 1.2 | 1.4 | 0.8 | 0.9 | 2.8 | 2.9 | 0.4 |
|  | **Total score** | **M** | **2.68** | **2.86** | **2.71** | **2.53** | **2.76** | **2.86** | **2.48** |
|  |  | **SD** | **1.26** | **1.32** | **1.27** | **1.29** | **1.29** | **1.46** | **1.05** |
| **Hyperactivity** | Restless, overactive, cannot stay still for long | Not true | 33.1 | 27.1 | 32.3 | 28.0 | 29.6 | 28.6 | 43.0 |
|  |  | Somewhat true | 41.0 | 36.2 | 44.6 | 58.6 | 38.0 | 42.9 | 37.4 |
|  |  | Certainly true | 25.9 | 36.7 | 23.1 | 13.4 | 32.4 | 28.6 | 19.7 |
|  | Constantly fidgeting or squirming | Not true | 42.4 | 37.2 | 67.2 | 34.2 | 39.3 | 39.1 | 46.6 |
|  |  | Somewhat true | 37.8 | 37.2 | 21.1 | 51.8 | 35.5 | 41.4 | 35.7 |
|  |  | Certainly true | 19.7 | 25.6 | 11.7 | 14.0 | 25.2 | 19.5 | 17.7 |
|  | Easily distracted, concentration wanders | Not true | 33.4 | 17.2 | 41.1 | 19.3 | 27.5 | 29.5 | 54.7 |
|  |  | Somewhat true | 44.9 | 47.1 | 41.1 | 66.2 | 45.9 | 46.7 | 34.3 |
|  |  | Certainly true | 21.6 | 35.7 | 17.8 | 14.5 | 26.6 | 23.8 | 11.0 |
|  | Thinks things out before acting | Not true | 17.5 | 10.1 | 28.6 | 21.7 | 11.9 | 16.4 | 21.4 |
|  |  | Somewhat true | 64.0 | 62.9 | 59.5 | 69.5 | 67.0 | 68.3 | 61.7 |
|  |  | Certainly true | 18.6 | 27.1 | 11.9 | 8.9 | 21.1 | 15.4 | 17.0 |
|  | Sees tasks through to the end, good attention span | Not true | 26.9 | 15.0 | 24.4 | 26.3 | 23.2 | 23.6 | 40.4 |
|  |  | Somewhat true | 55.3 | 54.9 | 57.5 | 63.2 | 59.3 | 60.1 | 49.5 |
|  |  | Certainly true | 17.7 | 30.0 | 18.1 | 10.5 | 17.6 | 16.4 | 10.1 |
|  | **Total score** | **M** | **4.67** | **4.85** | **4.37** | **4.91** | **4.89** | **4.84** | **4.39** |
|  |  | **SD** | **1.60** | **1.64** | **1.49** | **1.25** | **1.72** | **1.80** | **1.55** |
| **Peer problems** | Rather solitary, tends to play alone | Not true | 56.3 | 50.3 | 68.2 | 41.6 | 57.8 | 42.4 | 69.6 |
|  |  | Somewhat true | 33.2 | 34.4 | 25.6 | 47.4 | 32.1 | 44.3 | 24.2 |
|  |  | Certainly true | 10.6 | 15.3 | 6.2 | 11.1 | 10.1 | 13.3 | 6.2 |
|  | Has at least one good friend | Not true | 65.7 | 53.3 | 56.7 | 63.3 | 66.1 | 55.8 | 83.2 |
|  |  | Somewhat true | 25.1 | 30.8 | 26.8 | 31.0 | 28.4 | 33.7 | 13.4 |
|  |  | Certainly true | 9.2 | 15.9 | 16.5 | 5.7 | 5.5 | 10.6 | 3.4 |
|  | Generally liked by other children | Not true | 71.4 | 68.3 | 76.8 | 46.5 | 68.5 | 68.4 | 84.7 |
|  |  | Somewhat true | 26.8 | 29.3 | 20.8 | 50.4 | 30.6 | 28.7 | 14.7 |
|  |  | Certainly true | 1.8 | 2.4 | 2.4 | 3.0 | 0.9 | 2.9 | 0.5 |
|  | Picked on or bullied by other children | Not true | 85.0 | 88.6 | 91.3 | 63.2 | 85.3 | 86.6 | 88.6 |
|  |  | Somewhat true | 13.2 | 9.6 | 7.1 | 34.2 | 11.9 | 11.0 | 10.3 |
|  |  | Certainly true | 1.8 | 1.8 | 1.6 | 2.6 | 2.8 | 2.4 | 1.1 |
|  | Gets on better with adults than with other children | Not true | 54.3 | 51.6 | 58.4 | 27.0 | 63.3 | 51.9 | 65.9 |
|  |  | Somewhat true | 35.0 | 36.1 | 30.4 | 54.4 | 32.1 | 35.1 | 27.7 |
|  |  | Certainly true | 10.7 | 12.4 | 11.2 | 18.6 | 4.6 | 13.0 | 6.4 |
|  | **Total score** | **M** | **4.54** | **4.44** | **4.14** | **5.02** | **4.40** | **4.60** | **4.54** |
|  |  | **SD** | **1.29** | **1.42** | **1.25** | **1.33** | **1.20** | **1.43** | **1.06** |
| **Prosocial behaviour** | Considerate of other people's feelings | Not true | 5.1 | 9.9 | 2.3 | 4.7 | 6.4 | 4.3 | 1.8 |
|  |  | Somewhat true | 44.8 | 50.9 | 33.1 | 70.3 | 45.0 | 50.0 | 29.7 |
|  |  | Certainly true | 50.0 | 39.3 | 64.6 | 25.0 | 48.6 | 45.7 | 68.6 |
|  | Shares readily with other children (treats, toys, pencils, e.) | Not true | 8.6 | 12.8 | 8.9 | 4.4 | 8.3 | 9.7 | 6.2 |
|  |  | Somewhat true | 50.0 | 54.3 | 51.2 | 56.8 | 52.3 | 50.7 | 42.6 |
|  |  | Certainly true | 41.4 | 33.0 | 39.8 | 38.9 | 39.5 | 39.6 | 51.2 |
|  | Helpful if someone is hurt, upset or feeling ill | Not true | 5.2 | 6.5 | 3.1 | 11.5 | 4.6 | 3.8 | 2.7 |
|  |  | Somewhat true | 33.8 | 31.6 | 35.2 | 41.2 | 35.8 | 39.1 | 30.1 |
|  |  | Certainly true | 61.0 | 61.9 | 61.7 | 47.4 | 59.6 | 57.1 | 67.2 |
|  | Kind to younger children | Not true | 3.2 | 4.6 | 3.1 | 5.2 | 2.8 | 3.9 | 0.9 |
|  |  | Somewhat true | 26.2 | 30.1 | 22.7 | 35.4 | 29.6 | 31.7 | 17.1 |
|  |  | Certainly true | 70.6 | 65.3 | 74.2 | 59.4 | 67.6 | 64.4 | 82.1 |
|  | Often volunteers to help others (parents, teachers, other children) | Not true | 8.6 | 13.2 | 12.7 | 3.9 | 8.3 | 13.9 | 3.5 |
|  |  | Somewhat true | 46.7 | 52.0 | 47.6 | 54.6 | 47.7 | 47.9 | 37.9 |
|  |  | Certainly true | 44.7 | 34.8 | 39.7 | 41.5 | 44.0 | 38.3 | 58.6 |
|  | **Total score** | **M** | **7.38** | **6.90** | **7.57** | **6.85** | **7.30** | **7.08** | **8.10** |
|  |  | **SD** | **2.11** | **2.23** | **1.90** | **1.85** | **2.12** | **2.04** | **1.96** |
| **SDQ** | **Total difficulties score** | **M** | **17.07** | **18.29** | **16.07** | **18.22** | **17.02** | **17.95** | **15.52** |
|  |  | **SD** | **5.03** | **5.35** | **4.49** | **3.90** | **4.47** | **5.38** | **4.74** |

Table S2. *Strengths and Difficulties Questionnaire Factor Models in the Overall Sample and Across Sites*

| Model | CFI | TLI | RMSEA [90% CI] | χ^2^ | df |
| --- | --- | --- | --- | --- | --- |
| (a) Six-factor model | | | | | |
| UK | 0.965 | 0.957 | 0.044 [0.037, 0.050] | 374.918 | 189 |
| ITA | 0.921 | 0.903 | 0.056 [0.039, 0.071] | 265.168 | 189 |
| CHN | 0.924 | 0.908 | 0.051 [0.040, 0.062] | 304.792 | 189 |
| AUS | 0.925 | 0.908 | 0.056 [0.036, 0.073] | 252.825 | 189 |
| USA | 0.928 | 0.912 | 0.053 [0.041, 0.064] | 299.739 | 189 |
| SWE | 0.968 | 0.961 | 0.051 [0.045, 0.056] | 462.889 | 189 |
| **Total** | **0.959** | **0.950** | **0.049 [0.046, 0.052]** | **998.912** | **189** |
| (b) Five-Factor Model | | | | | |
| UK | 0.907 | 0.892 | 0.070 [0.064, 0.075] | 693.959 | 199 |
| ITA | 0.893 | 0.875 | 0.063 [0.048, 0.077] | 302.024 | 199 |
| CHN | 0.900 | 0.884 | 0.057 [0.048, 0.067] | 352.523 | 199 |
| AUS | 0.886 | 0.868 | 0.067 [0.050, 0.082] | 295.598 | 199 |
| USA | 0.859 | 0.837 | 0.072 [0.062, 0.081] | 414.217 | 199 |
| SWE | 0.941 | 0.932 | 0.067 [0.062, 0.072] | 706.054 | 199 |
| **Total** | **0.919** | **0.906** | **0.068 [0.065, 0.070]** | **1799.349** | **199** |
| (c) Five-Factor with One Higher-Order Factor Model | | | | | |
| UK | 0.892 | 0.878 | 0.074 [0.069, 0.080] | 776.782 | 204 |
| ITA | 0.885 | 0.869 | 0.065 [0.050, 0.078] | 314.773 | 204 |
| CHN | 0.859 | 0.840 | 0.067 [0.058, 0.076] | 419.997 | 204 |
| AUS | 0.883 | 0.868 | 0.067 [0.050, 0.082] | 302.924 | 204 |
| USA | 0.845 | 0.825 | 0.074 [0.065, 0.084] | 440.892 | 204 |
| SWE | 0.941 | 0.933 | 0.067 [0.061, 0.072] | 716.611 | 204 |
| **Total** | **0.905** | **0.892** | **0.072 [0.070, 0.075]** | **2085.290** | **204** |
| (d) Three-Factor Model | | | | | |
| UK | 0.833 | 0.812 | 0.092 [0.087, 0.097] | 1096.483 | 206 |
| ITA | 0.870 | 0.855 | 0.068 [0.054, 0.082] | 330.462 | 206 |
| CHN | 0.829 | 0.808 | 0.074 [0.065, 0.083] | 468.441 | 206 |
| AUS | 0.800 | 0.776 | 0.087 [0.073, 0.101] | 375.554 | 206 |
| USA | 0.806 | 0.783 | 0.083 [0.074, 0.092] | 502.179 | 206 |
| SWE | 0.906 | 0.895 | 0.083 [0.078, 0.088] | 1014.968 | 206 |
| **Total** | **0.860** | **0.843** | **0.087 [0.085, 0.090]** | **2973.580** | **206** |
| (e) Two-Factor Model | | | | | |
| UK | 0.755 | 0.728 | 0.111 [0.105, 0.116] | 1509.906 | 208 |
| ITA | 0.837 | 0.818 | 0.076 [0.063, 0.089] | 364.841 | 208 |
| CHN | 0.798 | 0.776 | 0.080 [0.071, 0.088] | 516.643 | 208 |
| AUS | 0.697 | 0.664 | 0.106 [0.093, 0.119] | 464.485 | 208 |
| USA | 0.760 | 0.734 | 0.092 [0.083, 0.101] | 574.899 | 208 |
| SWE | 0.823 | 0.803 | 0.114 [0.109, 0.119] | 1735.734 | 208 |
| **Total** | **0.785** | **0.761** | **0.108 [0.105, 0.110]** | **4449.163** | **208** |
| (f) Single-Factor Model | | | | | |
| UK | 0.709 | 0.678 | 0.120 [0.115, 0.126] | 1759.722 | 209 |
| ITA | 0.791 | 0.769 | 0.086 [0.073, 0.098] | 409.319 | 209 |
| CHN | 0.783 | 0.760 | 0.082 [0.074, 0.091] | 541.043 | 209 |
| AUS | 0.656 | 0.619 | 0.113 [0.101, 0.126] | 500.985 | 209 |
| USA | 0.714 | 0.684 | 0.100 [.0.091, 0.109] | 647.030 | 209 |
| SWE | 0.751 | 0.725 | 0.135 [0.130, 0.140] | 2356.515 | 209 |
| **Total** | **0.739** | **0.711** | **0.118 [0.116, 0.121]** | **5365.374** | **209** |

*Note*. CFI = Cumulative Fit Index; TLI = Tucker Lewis Index; RMSEA = Root Mean Square Error of Approximation.

Table S3. *Factorial Loadings and Factors Correlations for the Five Factor Model Across Sites*

| Model Parameter | Unstandardized Estimate | SE | Two-Tailed  *p*-value |
| --- | --- | --- | --- |
|  |  |  |  |
| *Model UK* |  |  |  |
|  |  |  |  |
| Emotional problems BY |  |  |  |
| Complains | 0.599 | 0.050 | 0.000 |
| Worries | 0.800 | 0.029 | 0.000 |
| Unhappy | 0.798 | 0.036 | 0.000 |
| Nervous | 0.713 | 0.039 | 0.000 |
| Fear | 0.800 | 0.036 | 0.000 |
|  |  |  |  |
| Conduct problems BY |  |  |  |
| Hot tempers | 0.694 | 0.036 | 0.000 |
| Obedient | 0.690 | 0.040 | 0.000 |
| Lies | 0.466 | 0.049 | 0.000 |
|  |  |  |  |
| Hyperactivity BY |  |  |  |
| Restless | 0.783 | 0.030 | 0.000 |
| Fidgeting | 0.796 | 0.028 | 0.000 |
| Distracted | 0.790 | 0.026 | 0.000 |
| Thinks before acting | 0.639 | 0.039 | 0.000 |
| Good attention | 0.730 | 0.033 | 0.000 |
|  |  |  |  |
| Peer problems BY |  |  |  |
| Solitary | 0.455 | 0.057 | 0.000 |
| Good friend | 0.731 | 0.044 | 0.000 |
| Liked by peers | 0.839 | 0.047 | 0.000 |
| Better with adults | 0.387 | 0.063 | 0.000 |
|  |  |  |  |
| Prosocial Behaviour BY |  |  |  |
| Considerate feelings | 0.822 | 0.029 | 0.000 |
| Shares | 0.690 | 0.038 | 0.000 |
| Helps | 0.750 | 0.034 | 0.000 |
| Kind | 0.676 | 0.042 | 0.000 |
| Volunteers | 0.661 | 0.036 | 0.000 |
|  |  |  |  |
| Conduct problems WITH |  |  |  |
| Emotional problems | 0.607 | 0.051 | 0.000 |
|  |  |  |  |
| Hyperactivity WITH |  |  |  |
| Emotional problems | 0.458 | 0.046 | 0.000 |
| Conduct problems | 0.784 | 0.040 | 0.000 |
|  |  |  |  |
| Peer problems WITH |  |  |  |
| Emotional problems | 0.353 | 0.054 | 0.000 |
| Conduct problems | 0.409 | 0.056 | 0.000 |
| Hyperactivity | 0.283 | 0.054 | 0.000 |
|  |  |  |  |
| Prosocial Behaviour WITH |  |  |  |
| Emotional problems | -0.335 | 0.053 | 0.000 |
| Conduct problems | -0.713 | 0.042 | 0.000 |
| Hyperactivity | -0.494 | 0.041 | 0.000 |
| Peer problems | -0.652 | 0.047 | 0.000 |
|  |  |  |  |
| *Model ITALY* |  |  |  |
|  |  |  |  |
| Emotional problems BY |  |  |  |
| Complains | 0.505 | 0.107 | 0.000 |
| Worries | 0.623 | 0.095 | 0.000 |
| Unhappy | 0.924 | 0.077 | 0.000 |
| Nervous | 0.666 | 0.073 | 0.000 |
| Fear | 0.708 | 0.068 | 0.000 |
|  |  |  |  |
| Conduct problems BY |  |  |  |
| Hot tempers | 0.598 | 0.073 | 0.000 |
| Obedient | 0.586 | 0.074 | 0.000 |
| Lies | 0.613 | 0.075 | 0.000 |
|  |  |  |  |
| Hyperactivity BY |  |  |  |
| Restless | 0.717 | 0.048 | 0.000 |
| Fidgeting | 0.807 | 0.065 | 0.000 |
| Distracted | 0.658 | 0.061 | 0.000 |
| Thinks before acting | 0.682 | 0.055 | 0.000 |
| Good attention | 0.694 | 0.058 | 0.000 |
|  |  |  |  |
| Peer problems BY |  |  |  |
| Solitary | 0.447 | 0.107 | 0.000 |
| Good friend | 0.667 | 0.097 | 0.000 |
| Liked by peers | 0.860 | 0.101 | 0.000 |
| Better with adults | 0.341 | 0.122 | 0.005 |
|  |  |  |  |
| Prosocial Behaviour BY |  |  |  |
| Considerate feelings | 0.872 | 0.071 | 0.000 |
| Shares | 0.678 | 0.090 | 0.000 |
| Helps | 0.641 | 0.077 | 0.000 |
| Kind | 0.517 | 0.103 | 0.000 |
| Volunteers | 0.636 | 0.084 | 0.000 |
|  |  |  |  |
| Conduct problems WITH |  |  |  |
| Emotional problems | 0.784 | 0.082 | 0.000 |
|  |  |  |  |
| Hyperactivity WITH |  |  |  |
| Emotional problems | 0.602 | 0.092 | 0.000 |
| Conduct problems | 1.085 | 0.076 | 0.000 |
|  |  |  |  |
| Peer problems WITH |  |  |  |
| Emotional problems | 0.579 | 0.107 | 0.000 |
| Conduct problems | 0.633 | 0.147 | 0.000 |
| Hyperactivity | 0.519 | 0.101 | 0.000 |
|  |  |  |  |
| Prosocial Behaviour WITH |  |  |  |
| Emotional problems | -0.317 | 0.100 | 0.001 |
| Conduct problems | -0.705 | 0.092 | 0.000 |
| Hyperactivity | -0.574 | 0.079 | 0.000 |
| Peer problems | -0.638 | 0.103 | 0.000 |
|  |  |  |  |
| *Model CHINA* |  |  |  |
|  |  |  |  |
| Emotional problems BY |  |  |  |
| Complains | 0.591 | 0.084 | 0.000 |
| Worries | 0.457 | 0.089 | 0.000 |
| Unhappy | 0.678 | 0.072 | 0.000 |
| Nervous | 0.497 | 0.068 | 0.000 |
| Fear | 0.391 | 0.073 | 0.000 |
|  |  |  |  |
| Conduct problems BY |  |  |  |
| Hot tempers | 0.718 | 0.055 | 0.000 |
| Obedient | 0.643 | 0.061 | 0.000 |
| Lies | 0.683 | 0.068 | 0.000 |
|  |  |  |  |
| Hyperactivity BY |  |  |  |
| Restless | 0.554 | 0.059 | 0.000 |
| Fidgeting | 0.673 | 0.050 | 0.000 |
| Distracted | 0.762 | 0.043 | 0.000 |
| Thinks before acting | 0.754 | 0.054 | 0.000 |
| Good attention | 0.794 | 0.045 | 0.000 |
|  |  |  |  |
| Peer problems BY |  |  |  |
| Solitary | 0.197 | 0.088 | 0.025 |
| Good friend | 0.569 | 0.081 | 0.000 |
| Liked by peers | 0.764 | 0.074 | 0.000 |
| Better with adults | -0.089 | 0.087 | 0.306 |
|  |  |  |  |
| Prosocial Behaviour BY |  |  |  |
| Considerate feelings | 0.752 | 0.057 | 0.000 |
| Shares | 0.565 | 0.052 | 0.000 |
| Helps | 0.420 | 0.063 | 0.000 |
| Kind | 0.611 | 0.055 | 0.000 |
| Volunteers | 0.651 | 0.058 | 0.000 |
|  |  |  |  |
| Conduct problems WITH |  |  |  |
| Emotional problems | 0.880 | 0.071 | 0.000 |
|  |  |  |  |
| Hyperactivity WITH |  |  |  |
| Emotional problems | 0.521 | 0.074 | 0.000 |
| Conduct problems | 0.646 | 0.066 | 0.000 |
|  |  |  |  |
| Peer problems WITH |  |  |  |
| Emotional problems | 0.549 | 0.117 | 0.000 |
| Conduct problems | 0.271 | 0.112 | 0.016 |
| Hyperactivity | 0.225 | 0.086 | 0.009 |
|  |  |  |  |
| Prosocial Behaviour WITH |  |  |  |
| Emotional problems | -0.511 | 0.090 | 0.000 |
| Conduct problems | -0.646 | 0.076 | 0.000 |
| Hyperactivity | -0.584 | 0.060 | 0.000 |
| Peer problems | -0.897 | 0.077 | 0.000 |
|  |  |  |  |
| *Model AUSTRALIA* |  |  |  |
|  |  |  |  |
| Emotional problems BY |  |  |  |
| Complains | 0.443 | 0.124 | 0.000 |
| Worries | 0.778 | 0.081 | 0.000 |
| Unhappy | 0.935 | 0.082 | 0.000 |
| Nervous | 0.575 | 0.094 | 0.000 |
| Fear | 0.694 | 0.086 | 0.000 |
|  |  |  |  |
| Conduct problems BY |  |  |  |
| Hot tempers | 0.569 | 0.092 | 0.000 |
| Obedient | 0.799 | 0.090 | 0.000 |
| Lies | 0.644 | 0.108 | 0.000 |
|  |  |  |  |
| Hyperactivity BY |  |  |  |
| Restless | 0.782 | 0.052 | 0.000 |
| Fidgeting | 0.811 | 0.049 | 0.000 |
| Distracted | 0.750 | 0.060 | 0.000 |
| Thinks before acting | 0.716 | 0.080 | 0.000 |
| Good attention | 0.760 | 0.078 | 0.000 |
|  |  |  |  |
| Peer problems BY |  |  |  |
| Solitary | 0.335 | 0.122 | 0.006 |
| Good friend | 0.720 | 0.082 | 0.000 |
| Liked by peers | 1.012 | 0.067 | 0.000 |
| Better with adults | 0.197 | 0.130 | 0.130 |
|  |  |  |  |
| Prosocial Behaviour BY |  |  |  |
| Considerate feelings | 0.832 | 0.064 | 0.000 |
| Shares | 0.659 | 0.080 | 0.000 |
| Helps | 0.672 | 0.085 | 0.000 |
| Kind | 0.787 | 0.064 | 0.000 |
| Volunteers | 0.689 | 0.069 | 0.000 |
|  |  |  |  |
| Conduct problems WITH |  |  |  |
| Emotional problems | 0.430 | 0.114 | 0.000 |
|  |  |  |  |
| Hyperactivity WITH |  |  |  |
| Emotional problems | 0.292 | 0.092 | 0.001 |
| Conduct problems | 0.570 | 0.098 | 0.000 |
|  |  |  |  |
| Peer problems WITH |  |  |  |
| Emotional problems | 0.394 | 0.125 | 0.002 |
| Conduct problems | 0.509 | 0.126 | 0.000 |
| Hyperactivity | 0.259 | 0.108 | 0.017 |
|  |  |  |  |
| Prosocial Behaviour WITH |  |  |  |
| Emotional problems | -0.385 | 0.103 | 0.000 |
| Conduct problems | -0.661 | 0.089 | 0.000 |
| Hyperactivity | -0.343 | 0.095 | 0.000 |
| Peer problems | -0.776 | 0.077 | 0.000 |
|  |  |  |  |
| *Model USA* |  |  |  |
|  |  |  |  |
| Emotional problems BY |  |  |  |
| Complains | 0.578 | 0.078 | 0.000 |
| Worries | 0.752 | 0.065 | 0.000 |
| Unhappy | 0.794 | 0.059 | 0.000 |
| Nervous | 0.697 | 0.059 | 0.000 |
| Fear | 0.659 | 0.060 | 0.000 |
|  |  |  |  |
| Conduct problems BY |  |  |  |
| Hot tempers | 0.619 | 0.069 | 0.000 |
| Obedient | 0.760 | 0.063 | 0.000 |
| Lies | 0.483 | 0.076 | 0.000 |
|  |  |  |  |
| Hyperactivity BY |  |  |  |
| Restless | 0.807 | 0.040 | 0.000 |
| Fidgeting | 0.789 | 0.047 | 0.000 |
| Distracted | 0.804 | 0.043 | 0.000 |
| Thinks before acting | 0.359 | 0.082 | 0.000 |
| Good attention | 0.555 | 0.071 | 0.000 |
|  |  |  |  |
| Peer problems BY |  |  |  |
| Solitary | 0.347 | 0.085 | 0.000 |
| Good friend | 0.624 | 0.080 | 0.000 |
| Liked by peers | 0.818 | 0.072 | 0.000 |
| Better with adults | 0.336 | 0.088 | 0.000 |
|  |  |  |  |
| Prosocial Behaviour BY |  |  |  |
| Considerate feelings | 0.802 | 0.055 | 0.000 |
| Shares | 0.679 | 0.072 | 0.000 |
| Helps | 0.631 | 0.062 | 0.000 |
| Kind | 0.644 | 0.072 | 0.000 |
| Volunteers | 0.516 | 0.079 | 0.000 |
|  |  |  |  |
| Conduct problems WITH |  |  |  |
| Emotional problems | 0.638 | 0.070 | 0.000 |
|  |  |  |  |
| Hyperactivity WITH |  |  |  |
| Emotional problems | 0.524 | 0.062 | 0.000 |
| Conduct problems | 0.684 | 0.081 | 0.000 |
|  |  |  |  |
| Peer problems WITH |  |  |  |
| Emotional problems | 0.567 | 0.081 | 0.000 |
| Conduct problems | 0.572 | 0.101 | 0.000 |
| Hyperactivity | 0.410 | 0.090 | 0.000 |
|  |  |  |  |
| Prosocial Behaviour WITH |  |  |  |
| Emotional problems | -0.376 | 0.086 | 0.000 |
| Conduct problems | -0.662 | 0.071 | 0.000 |
| Hyperactivity | -0.334 | 0.079 | 0.000 |
| Peer problems | -0.742 | 0.069 | 0.000 |
|  |  |  |  |
| *Model SWEDEN* |  |  |  |
|  |  |  |  |
| Emotional problems BY |  |  |  |
| Complains | 0.516 | 0.054 | 0.000 |
| Worries | 0.799 | 0.032 | 0.000 |
| Unhappy | 0.810 | 0.040 | 0.000 |
| Nervous | 0.759 | 0.036 | 0.000 |
| Fear | 0.820 | 0.032 | 0.000 |
|  |  |  |  |
| Conduct problems BY |  |  |  |
| Hot tempers | 0.743 | 0.031 | 0.000 |
| Obedient | 0.796 | 0.031 | 0.000 |
| Lies | 0.595 | 0.048 | 0.000 |
|  |  |  |  |
| Hyperactivity BY |  |  |  |
| Restless | 0.917 | 0.015 | 0.000 |
| Fidgeting | 0.903 | 0.015 | 0.000 |
| Distracted | 0.860 | 0.019 | 0.000 |
| Thinks before acting | 0.649 | 0.035 | 0.000 |
| Good attention | 0.778 | 0.026 | 0.000 |
|  |  |  |  |
| Peer problems BY |  |  |  |
| Solitary | 0.656 | 0.056 | 0.000 |
| Good friend | 0.556 | 0.065 | 0.000 |
| Liked by peers | 0.950 | 0.056 | 0.000 |
| Better with adults | 0.460 | 0.059 | 0.000 |
|  |  |  |  |
| Prosocial Behaviour BY |  |  |  |
| Considerate feelings | 0.862 | 0.026 | 0.000 |
| Shares | 0.751 | 0.031 | 0.000 |
| Helps | 0.859 | 0.025 | 0.000 |
| Kind | 0.653 | 0.042 | 0.000 |
| Volunteers | 0.815 | 0.027 | 0.000 |
|  |  |  |  |
| Conduct problems WITH |  |  |  |
| Emotional problems | 0.446 | 0.052 | 0.000 |
|  |  |  |  |
| Hyperactivity WITH |  |  |  |
| Emotional problems | 0.323 | 0.047 | 0.000 |
| Conduct problems | 0.816 | 0.031 | 0.000 |
|  |  |  |  |
| Peer problems WITH |  |  |  |
| Emotional problems | 0.397 | 0.057 | 0.000 |
| Conduct problems | 0.400 | 0.061 | 0.000 |
| Hyperactivity | 0.429 | 0.052 | 0.000 |
|  |  |  |  |
| Prosocial Behaviour WITH |  |  |  |
| Emotional problems | -0.268 | 0.051 | 0.000 |
| Conduct problems | -0.632 | 0.043 | 0.000 |
| Hyperactivity | -0.456 | 0.042 | 0.000 |
| Peer problems | -0.518 | 0.048 | 0.000 |

Table S4. *Factorial Loadings, Items Thresholds and (Unconstrained) Latent Means for the Final Partially Invariant Five Factor Model*

| Model Parameter |  | Unstandardized Estimate | SE | Two-Tailed  p-value |
| --- | --- | --- | --- | --- |
|  |  |  |  |  |
| **Factorial loadings** |  |  |  |  |
|  |  |  |  |  |
| Emotional problems BY | |  |  |  |
| Complains | | 0.562 | 0.039 | 0.000 |
| Worries | | 0.797 | 0.026 | 0.000 |
| Unhappy | | 0.809 | 0.031 | 0.000 |
| Nervous | | 0.698 | 0.034 | 0.000 |
| Fear | | 0.784 | 0.031 | 0.000 |
|  | |  |  |  |
| Conduct problems BY | |  |  |  |
| Hot tempers | | 0.688 | 0.030 | 0.000 |
| Obedient | | 0.699 | 0.032 | 0.000 |
| Lies | | 0.504 | 0.036 | 0.000 |
|  | |  |  |  |
| Hyperactivity BY | |  |  |  |
| Restless | | 0.773 | 0.027 | 0.000 |
| Fidgeting | | 0.793 | 0.025 | 0.000 |
| Distracted | | 0.778 | 0.023 | 0.000 |
| Thinks before acting | | 0.648 | 0.030 | 0.000 |
| Good attention | | 0.735 | 0.028 | 0.000 |
|  | |  |  |  |
| Peer problems BY | |  |  |  |
| Solitary | | 0.398 | 0.040 | 0.000 |
| Good friend | | 0.654 | 0.035 | 0.000 |
| Liked by peers | | 0.889 | 0.039 | 0.000 |
|  | |  |  |  |
| Prosocial Behaviour BY | |  |  |  |
| Considerate feelings | | 0.806 | 0.025 | 0.000 |
| Shares | | 0.685 | 0.032 | 0.000 |
| Helps | | 0.754 | 0.029 | 0.000 |
| Kind | | 0.667 | 0.034 | 0.000 |
| Volunteers | | 0.668 | 0.030 | 0.000 |
|  | |  |  |  |
| **Item thresholds** | |  |  |  |
| Complains | 1 | 0.387 | 0.044 | 0.000 |
|  | 2 | 1.422 | 0.073 | 0.000 |
| Worries | 1 | -0.004 | 0.047 | 0.940 |
|  | 2 | 1.149 | 0.061 | 0.000 |
| Unhappy | 1 | 0.271 | 0.048 | 0.000 |
|  | 2 | 1.396 | 0.069 | 0.000 |
| Nervous | 1 | -0.464 | 0.047 | 0.000 |
|  | 2 | 0.735 | 0.054 | 0.000 |
| Fear | 1 | -0.154 | 0.048 | 0.001 |
|  | 2 | 1.021 | 0.059 | 0.000 |
| Hot tempers | 1 | -0.558 | 0.049 | 0.000 |
|  | 2 | 0.532 | 0.050 | 0.000 |
| Obedient | 1 | -0.648 | 0.050 | 0.000 |
|  | 2 | 0.893 | 0.057 | 0.000 |
| Lies | 1 | 0.315 | 0.040 | 0.000 |
|  | 2 | 1.481 | 0.083 | 0.000 |
| Restless | 1 | -0.714 | 0.048 | 0.000 |
|  | 2 | 0.319 | 0.046 | 0.000 |
| Fidgeting | 1 | -0.495 | 0.046 | 0.000 |
|  | 2 | 0.523 | 0.048 | 0.000 |
| Distracted | 1^(a)^ | -0.957 | 0.054 | 0.000 |
|  | 2 | 0.494 | 0.047 | 0.000 |
| Thinks before acting | 1 | -1.195 | 0.059 | 0.000 |
|  | 2 | 0.659 | 0.051 | 0.000 |
| Good attention | 1 | -0.892 | 0.050 | 0.000 |
|  | 2 | 0.656 | 0.051 | 0.000 |
| Solitary | 1 | -0.011 | 0.038 | 0.779 |
|  | 2 | 1.043 | 0.070 | 0.000 |
| Good friend | 1 | 0.196 | 0.047 | 0.000 |
|  | 2 | 1.154 | 0.063 | 0.000 |
| Liked by peers | 1 | 0.303 | 0.057 | 0.000 |
|  | 2 | 1.973 | 0.097 | 0.000 |
| Considerate feelings | 1 | -1.397 | 0.065 | 0.000 |
|  | 2 | 0.226 | 0.047 | 0.000 |
| Shares | 1 | -1.160 | 0.061 | 0.000 |
|  | 2 | 0.421 | 0.047 | 0.000 |
| Helps | 1 | -1.538 | 0.071 | 0.000 |
|  | 2 | -0.069 | 0.046 | 0.128 |
| Kind | 1 | -1.744 | 0.083 | 0.000 |
|  | 2 | -0.373 | 0.045 | 0.000 |
| Volunteers | 1 | -1.152 | 0.061 | 0.000 |
|  | 2 | 0.323 | 0.043 | 0.000 |
|  | |  |  |  |
| **Latent means** | |  |  |  |
|  | |  |  |  |
| *Model UK* | |  |  |  |
|  | |  |  |  |
| Emotional problems | | 0.000 | 0.000 | - |
| Conduct problems | | 0.000 | 0.000 | - |
| Hyperactivity | | 0.000 | 0.000 | - |
| Peer problems | | 0.000 | 0.000 | - |
| Prosocial behaviour | | 0.000 | 0.000 | - |
|  | |  |  |  |
| *Model ITALY* | |  |  |  |
|  | |  |  |  |
| Emotional problems | | -0.387 | 0.135 | 0.004 |
| Conduct problems | | -0.254 | 0.142 | 0.073 |
| Hyperactivity | | -0.709 | 0.117 | 0.000 |
| Peer problems | | -0.490 | 0.170 | 0.004 |
| Prosocial behaviour | | 0.429 | 0.121 | 0.000 |
|  | |  |  |  |
| *Model CHINA* | |  |  |  |
|  | |  |  |  |
| Emotional problems | | -0.325 | 0.098 | 0.001 |
| Conduct problems | | -0.278 | 0.100 | 0.006 |
| Hyperactivity | | -0.519 | 0.088 | 0.000 |
| Peer problems | | 0.072 | 0.108 | 0.503 |
| Prosocial behaviour | | 0.046 | 0.094 | 0.624 |
|  | |  |  |  |
| *Model AUSTRALIA* | |  |  |  |
|  | |  |  |  |
| Emotional problems | | -0.394 | 0.139 | 0.005 |
| Conduct problems | | -0.262 | 0.146 | 0.073 |
| Hyperactivity | | -0.240 | 0.116 | 0.039 |
| Peer problems | | -0.265 | 0.145 | 0.067 |
| Prosocial behaviour | | 0.214 | 0.126 | 0.090 |
|  | |  |  |  |
| *Model USA* | |  |  |  |
|  | |  |  |  |
| Emotional problems | | -0.283 | 0.104 | 0.007 |
| Conduct problems | | -0.173 | 0.106 | 0.104 |
| Hyperactivity | | -0.345 | 0.091 | 0.000 |
| Peer problems | | -0.092 | 0.116 | 0.430 |
| Prosocial behaviour | | 0.178 | 0.100 | 0.074 |
|  | |  |  |  |
| *Model SWEDEN* | |  |  |  |
|  | |  |  |  |
| Emotional problems | | -0.482 | 0.076 | 0.000 |
| Conduct problems | | -0.538 | 0.082 | 0.000 |
| Hyperactivity | | -0.630 | 0.069 | 0.000 |
| Peer problems | | -1.027 | 0.115 | 0.000 |
| Prosocial behaviour | | 0.650 | 0.076 | 0.000 |

Note. Latent means are fixed to zero in the reference group only (i.e., the UK) for model identification requirements.

^(a)^ Noninvariant item threshold in the Sweden group only (i.e., Item 15, Threshold 1 = -0.429, SE = 0.059)

Table S5. *Factorial Loadings, Items Thresholds and (Partially Constrained) Latent Means for the Final Partially Invariant Five Factor Model*

| Model Parameter |  | Unstandardized Estimate | SE | Two-Tailed  *p*-value |
| --- | --- | --- | --- | --- |
|  |  |  |  |  |
| **Factorial loadings** |  |  |  |  |
|  |  |  |  |  |
| Emotional problems BY | |  |  |  |
| Complains | | 0.564 | 0.039 | 0.000 |
| Worries | | 0.797 | 0.026 | 0.000 |
| Unhappy | | 0.809 | 0.031 | 0.000 |
| Nervous | | 0.698 | 0.034 | 0.000 |
| Fear | | 0.783 | 0.031 | 0.000 |
|  | |  |  |  |
| Conduct problems BY | |  |  |  |
| Hot tempers | | 0.650 | 0.029 | 0.000 |
| Obedient | | 0.675 | 0.032 | 0.000 |
| Lies | | 0.468 | 0.032 | 0.000 |
|  | |  |  |  |
| Hyperactivity BY | |  |  |  |
| Restless | | 0.774 | 0.027 | 0.000 |
| Fidgeting | | 0.794 | 0.025 | 0.000 |
| Distracted | | 0.773 | 0.023 | 0.000 |
| Thinks before acting | | 0.650 | 0.030 | 0.000 |
| Good attention | | 0.736 | 0.028 | 0.000 |
|  | |  |  |  |
| Peer problems BY | |  |  |  |
| Solitary | | 0.388 | 0.039 | 0.000 |
| Good friend | | 0.643 | 0.034 | 0.000 |
| Liked by peers | | 0.875 | 0.039 | 0.000 |
|  | |  |  |  |
| Prosocial Behaviour BY | |  |  |  |
| Considerate feelings | | 0.798 | 0.025 | 0.000 |
| Shares | | 0.681 | 0.031 | 0.000 |
| Helps | | 0.748 | 0.028 | 0.000 |
| Kind | | 0.656 | 0.033 | 0.000 |
| Volunteers | | 0.662 | 0.029 | 0.000 |
|  | |  |  |  |
| **Item thresholds** | |  |  |  |
| Complains | 1 | 0.612 | 0.047 | 0.000 |
|  | 2 | 1.652 | 0.080 | 0.000 |
| Worries | 1 | 0.323 | 0.039 | 0.000 |
|  | 2 | 1.476 | 0.062 | 0.000 |
| Unhappy | 1 | 0.604 | 0.042 | 0.000 |
|  | 2 | 1.727 | 0.072 | 0.000 |
| Nervous | 1 | -0.174 | 0.034 | 0.000 |
|  | 2 | 1.024 | 0.054 | 0.000 |
| Fear | 1 | 0.174 | 0.037 | 0.000 |
|  | 2 | 1.347 | 0.061 | 0.000 |
| Hot tempers | 1 | -0.336 | 0.031 | 0.000 |
|  | 2 | 0.682 | 0.039 | 0.000 |
| Obedient | 1 | -0.428 | 0.033 | 0.000 |
|  | 2 | 1.040 | 0.049 | 0.000 |
| Lies | 1 | 0.428 | 0.034 | 0.000 |
|  | 2 | 1.503 | 0.082 | 0.000 |
| Restless | 1 | -0.291 | 0.033 | 0.000 |
|  | 2 | 0.737 | 0.040 | 0.000 |
| Fidgeting | 1 | -0.062 | 0.033 | 0.057 |
|  | 2 | 0.953 | 0.044 | 0.000 |
| Distracted | 1^(a)^ | -0.582 | 0.045 | 0.000 |
|  | 2 | 0.901 | 0.042 | 0.000 |
| Thinks before acting | 1 | -0.839 | 0.047 | 0.000 |
|  | 2 | 1.009 | 0.050 | 0.000 |
| Good attention | 1 | -0.492 | 0.037 | 0.000 |
|  | 2 | 1.049 | 0.048 | 0.000 |
| Solitary | 1 | 0.028 | 0.032 | 0.374 |
|  | 2 | 1.058 | 0.070 | 0.000 |
| Good friend | 1 | 0.231 | 0.035 | 0.000 |
|  | 2 | 1.165 | 0.059 | 0.000 |
| Liked by peers | 1 | 0.368 | 0.041 | 0.000 |
|  | 2 | 1.999 | 0.093 | 0.000 |
| Considerate feelings | 1 | -1.458 | 0.058 | 0.000 |
|  | 2 | 0.134 | 0.033 | 0.000 |
| Shares | 1 | -1.220 | 0.057 | 0.000 |
|  | 2 | 0.345 | 0.036 | 0.000 |
| Helps | 1 | -1.592 | 0.066 | 0.000 |
|  | 2 | -0.146 | 0.035 | 0.000 |
| Kind | 1 | -1.776 | 0.080 | 0.000 |
|  | 2 | -0.433 | 0.038 | 0.000 |
| Volunteers | 1 | -1.203 | 0.056 | 0.000 |
|  | 2 | 0.254 | 0.033 | 0.000 |
|  | |  |  |  |
| **Latent means** | |  |  |  |
|  | |  |  |  |
| *Model UK* | |  |  |  |
|  | |  |  |  |
| Emotional problems | | 0.412 | 0.065 | 0.000 |
| Conduct problems | | 0.000 | 0.000 | - |
| Hyperactivity | | 0.535 | 0.059 | 0.000 |
| Peer problems | | 0.000 | 0.000 | - |
| Prosocial behaviour | | 0.000 | 0.000 | - |
|  | |  |  |  |
| *Model ITALY* | |  |  |  |
|  | |  |  |  |
| Emotional problems | | 0.000 | 0.000 | - |
| Conduct problems | | 0.000 | 0.000 | - |
| Hyperactivity | | 0.000 | 0.000 | - |
| Peer problems | | 0.000 | 0.000 | - |
| Prosocial behaviour | | 0.000 | 0.000 | - |
|  | |  |  |  |
| *Model CHINA* | |  |  |  |
|  | |  |  |  |
| Emotional problems | | 0.000 | 0.000 | - |
| Conduct problems | | 0.000 | 0.000 | - |
| Hyperactivity | | 0.000 | 0.000 | - |
| Peer problems | | 0.000 | 0.000 | - |
| Prosocial behaviour | | 0.000 | 0.000 | - |
|  | |  |  |  |
| *Model AUSTRALIA* | |  |  |  |
|  | |  |  |  |
| Emotional problems | | 0.000 | 0.000 | - |
| Conduct problems | | 0.000 | 0.000 | - |
| Hyperactivity | | 0.000 | 0.000 | - |
| Peer problems | | 0.000 | 0.000 | - |
| Prosocial behaviour | | 0.000 | 0.000 | - |
|  | |  |  |  |
| *Model USA* | |  |  |  |
|  | |  |  |  |
| Emotional problems | | 0.000 | 0.000 | - |
| Conduct problems | | 0.000 | 0.000 | - |
| Hyperactivity | | 0.000 | 0.000 | - |
| Peer problems | | 0.000 | 0.000 | - |
| Prosocial behaviour | | 0.000 | 0.000 | - |
|  | |  |  |  |
| *Model SWEDEN* | |  |  |  |
|  | |  |  |  |
| Emotional problems | | 0.000 | 0.000 | - |
| Conduct problems | | 0.000 | 0.000 | - |
| Hyperactivity | | 0.000 | 0.000 | - |
| Peer problems | | -0.950 | 0.105 | 0.000 |
| Prosocial behaviour | | 0.547 | 0.065 | 0.000 |

*Note.* Equal means across sites are constrained to zero. Estimates for the unconstrained latent means scores (i.e., emotional problems and hyperactivity for the UK and peer problems and prosocial behaviour for Sweden) are interpreted as standard deviation from constrained means.

^(a)^ Noninvariant item threshold in the Sweden group only (i.e., Item 15, Threshold 1 = 0.105, SE = 0.047).
